# The Association Between Heart Rate Variability and 90-Day Prognosis in Patients With Transient Ischemic Attack and Minor Stroke

**DOI:** 10.3389/fneur.2021.636474

**Published:** 2021-05-28

**Authors:** Changhong Li, Xia Meng, Yuesong Pan, Zixiao Li, Mengxing Wang, Yongjun Wang

**Affiliations:** ^1^Department of Neurology, Beijing Tiantan Hospital, Capital Medical University, Beijing, China; ^2^Department of Neurology, Beijing Haidian Hospital, Beijing, China; ^3^China National Clinical Research Center for Neurological Diseases, Beijing, China; ^4^Center of Stroke, Beijing Institute for Brain Disorders, Beijing, China; ^5^Beijing Key Laboratory of Translational Medicine for Cerebrovascular Disease, Beijing, China

**Keywords:** neurological function, prognosis, stroke, transient ischemic attack, heart rate variability, stroke recurrence

## Abstract

**Background:** Low heart rate variability (HRV) is known to be associated with increased all-cause, cardiovascular, and cerebrovascular mortality but its association with clinical outcomes in patients with transient ischemic attack (TIA) or minor stroke is unclear.

**Methods:** We selected TIA and minor stroke patients from a prospective registration study. From each continuous electrocardiograph (ECG) record, each QRS complex was detected and normal-to-normal (N-N) intervals were determined. The standard deviation of all N-N intervals (SDNN) and the square root of the mean squared differences of successive N-N intervals (RMSSD) were calculated. Logistic regression analysis and Cox regression analysis were performed to assess the outcomes of patients at 90 days, and the odds and risk ratios (OR/HR) of each index quartile were compared.

**Results:** Compared with SDNN patients in the lowest quartile, neurological disability was significantly reduced in other quartile groups at 90 days, with significant differences [OR of group Q2 was 0.659; 95% confidence interval (CI), 0.482–0.900; *p* = 0.0088; OR of group Q3 was 0.662; 95% CI, 0.478–0.916; *p* = 0.0127; OR of group Q4 was 0.441; 95% CI, 0.305–0.639; *p* <0.0001]. Compared with the lowest quartile, the recurrence rate of TIA or minor stroke in patients of the two higher quartiles (Q3 and Q4) of SDNN was significantly reduced at 90 days (HR of Q3 group was 0.732; 95% CI, 0.539–0.995; *p* = 0.0461; HR of Q4 group was 0.528; 95% CI, 0.374–0.745; *p* = 0.0003).

**Conclusions:** Based on our findings, autonomic dysfunction is an adverse indicator for neurological function prognosis and stroke recurrence 90 days after TIA or minor stroke.

## Introduction

Stroke is the second leading cause of death worldwide (1) and the leading cause of mortality and disability in China (2). About 40% of stroke survivors are disabled [modified Rankin Scale (mRS) score 3–5] between 1 month and 5 years after stroke (3). Depending on the circumstances of treatment, the rate of stroke recurrence 90 days after the first ischemic event ranges from 3.7 to 20% (4–6). Approximately 40% of recurrent stroke events are fatal within 30 days, which is nearly twice the 30-day case fatality of a first-ever stroke (7). According to the data of the Third China National Stroke Registry (CNSR-III), TIA and minor stroke (an National Institutes of Health Stroke Scale (NIHSS) score ≤ 5) account for about 73% of acute ischemic stroke cases. Both TIA and minor stroke are characterized by a high risk of early stroke recurrence (8). Currently, assessment tools have limitations in predicting the early recurrence of stroke (9–12). It is still challenging to stratify the risk and identify high-risk patients accurately in the early treatment stage of stroke.

Heart rate variability (HRV) is a commonly used quantitative marker for measuring autonomic nerve system (13). HRV is easy to obtain. It quantifies sympathetic-vagus regulation at the sinus level as a tool (14) for assessing overall heart health and autonomic nerve system function (13, 15). Dysfunction of the autonomic nerve system after stroke increases the risk of stroke recurrence and death (16–18). Therefore, exploitation of the predictive function of HRV in risk stratification tools has become an important measure to identify high-risk populations. Although correlation between autonomic nerve system function and stroke prognosis has been studied previously, the sample sizes were small (19, 20).

To date, no study has been done to evaluate how HRV is related to a comprehensive 90-day prognosis in patients with TIA or minor stroke. Using the CNSR-III database, this study focused on the correlation between HRV and 90-day outcomes in patients with TIA and minor stroke including neurological disability, stroke recurrence, and cardiovascular death.

## Methods

### Study Population

The CNSR-III database is a nationwide prospective clinical registry of ischemic stroke or TIA in China based on etiology, imaging, and biology markers. The detailed study design of the CNSR-III trial has been described elsewhere (21). Briefly, between August 2015 and March 2018, the CNSR-III recruited consecutive patients with ischemic stroke or TIA from 201 hospitals that covered 22 provinces and four municipalities in China. Informed consent received from the patient or legally authorized representative (primarily spouse, parents, adult children, otherwise indicated). Clinical data were collected prospectively using an electronic data capture system by face-to-face interviews. Brain imaging, including brain magnetic resonance imaging (MRI) and computed tomography (CT), were completed at baseline. Blood samples were collected and biomarkers were tested at baseline. Face-to-face follow-up was conducted at 3 months, and telephone follow-up was conducted at 6 months and 1–5 years.

The registry recruited consecutive patients who met the following criteria: age >18 years; ischemic stroke or TIA; within 7 days from the onset of symptoms to enrolment; Acute ischemic stroke was diagnosed according to the World Health Organization (WHO) criteria (22) and confirmed by MRI or brain CT. Patients who had silent cerebral infarction with no manifestation of symptoms and signs or who refused to participate in the registry were excluded. The protocol of the CNSR-III trial was approved by the ethics committee at Beijing Tiantan Hospital affiliated to Capital Medical University (IRB Approval Number: KY2015-001-01) and all participating centers. In this study, minor stroke was defined as an NIHSS score ≤ 5.

**Figure 1:**
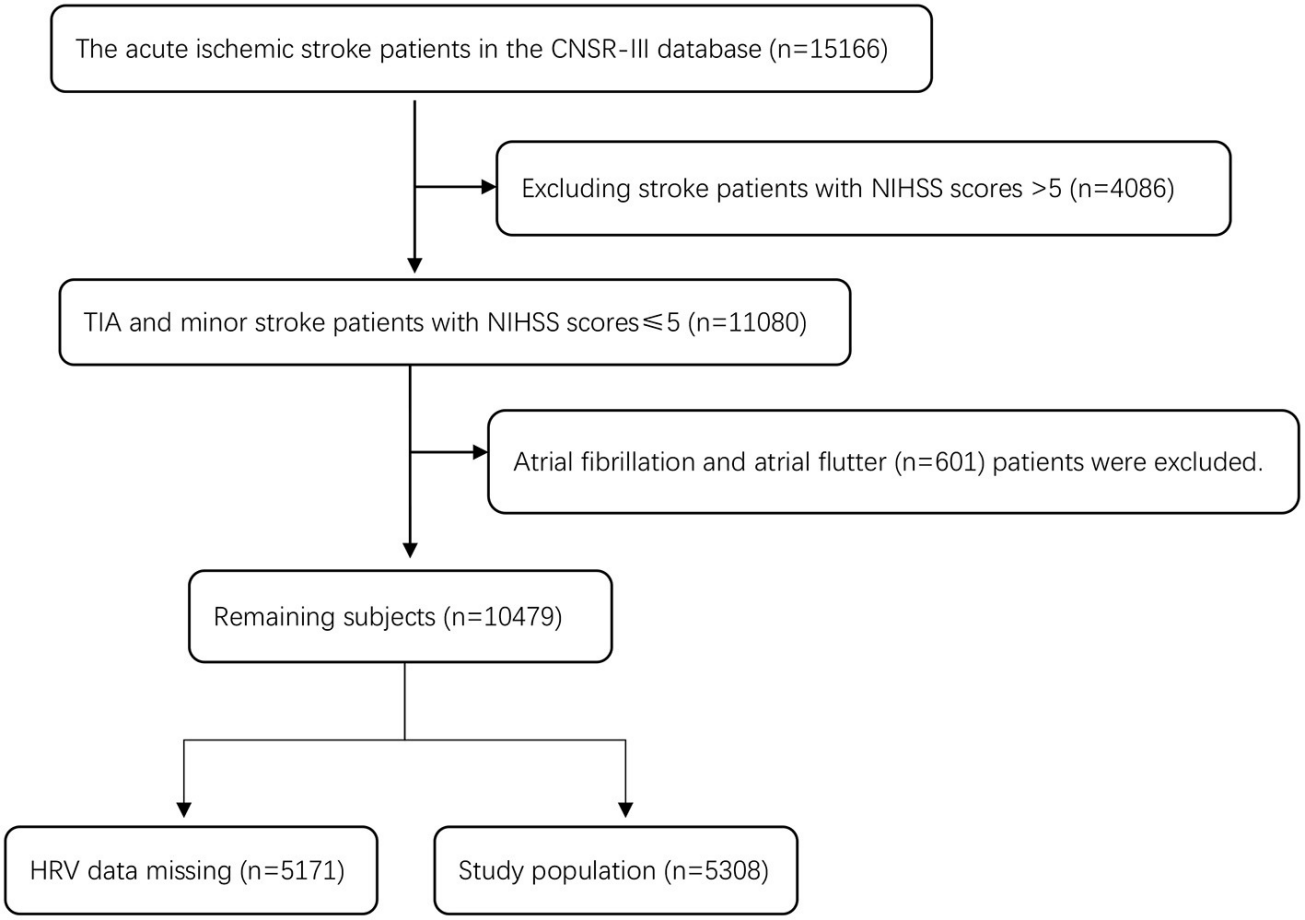
Flowchart of patient selection from CNSR-III database.

There were 15,166 patients in CNSR-III, and 4,086 patients with an NIHSS score >5 were excluded. There were 11,080 patients with TIA and minor stroke (an NIHSS score ≤ 5). Six hundred and one patients with atrial fibrillation and atrial flutter (*n* = 601) or those with missing HRV data (*n* = 5,171) were excluded (including no 24-h ECG examination or HRV data generated during 24-h ECG examination). A total of 5,308 patients were eligible for the study. Figure 1 shows a detailed flow chart for the study population selection from CNSR-III.

### Baseline Variables

Age, sex, smoking history (never, occasionally, current, and past), drinking history (never, occasionally, current, and past), body mass index (BMI), heart rate on admission, blood pressure on admission, NIHSS (National Institute of Health stroke scale), medical history (including stroke, heart disease, hypertension, diabetes mellitus, and hyperlipidemia), mRS score before onset, medication history, secondary prevention treatment, and stroke etiology were all collected at the baseline.

During hospitalization, the patient received 24-h ECG, from which the SDNN and RMSSD were automatically obtained. SDNN is a global index of HRV, and reflects the standard deviation of the normal R-R intervals (N-N intervals) (23). RMSSD is the square root of the mean squared differences of successive N-N intervals. It is thought to reflect the activity of the parasympathetic nervous system (13).

### Outcome Measures

Neurological function prognosis, stroke recurrence, and cardiovascular death were recorded 90 day after TIA or minor stroke. Disability after stroke was defined as an mRS score ≥ 3. Recurrent stroke was defined as new ischemic and recurrent hemorrhagic strokes (intracerebral and subarachnoid hemorrhages). Cardiovascular death was defined as ischemic stroke, hemorrhagic stroke, sudden cardiac death, acute myocardial infarction, death directly caused by heart failure, and other cardiovascular death [including cardiac arrhythmias unrelated to sudden cardiac death, pulmonary embolism, cardiovascular intervention (unrelated to acute myocardial infarction), aortic aneurysm rupture, and peripheral arterial disease]. Each case fatality was either confirmed on a death certificate from the attending hospital or local citizen registry.

### Statistical Analysis

Continuous variables are expressed as the mean ± standard deviation, and classification variables are expressed as a percentage. A quartile classification method was used for SDNN and RMSSD, with the lowest quartile as a reference for all comparisons. Baseline variables between different quartile groups were compared using a chi-square test for classification variables and Kruskal-wallis-test for continuous variables. Logistic regression analysis and Cox regression analysis were used to calculate the odds and risk ratios (OR/HR) and 95% confidence intervals (CIs). The adjusted clinical covariables included age, sex, smoking, alcohol consumption, previous stroke history, heart disease, hypertension, diabetes mellitus, lipid metabolic disorders, and other variables with a *p* < 0.1. A two-sided significance level of *P* < 0.05 was determined. All analyses were performed using SAS 9.4 software.

## Results

A total of 5,308 patients (mean age, 61.13 ± 10.81 years) were enrolled in the study: 69.11% males (mean age, 60.2 ± 10.8 years) and 30.89% females (mean age, 63.2 ± 10.4 years). SDNN was 108.32 ± 30.55 ms and RMSSD was 30.79 ± 15.05 ms. Among the patients, the prevalence of previous stroke/TIA, coronary heart disease, hypertension, diabetes mellitus, and dyslipidemia were 22.89% (1,215 cases), 10.70% (568 cases), 62.08% (3,296 cases), 22.92% (1,217 cases), and 8.16% (433 cases), respectively. The proportion of anti-platelet, anticoagulation, stains, antioxidant, hypoglycemic and antihypertensive drugs in secondary prevention were 97.98% (5,201 cases), 5.58% (296 cases), 95.65% (5,077 cases), 15.79% (838 cases), 24.89% (1,321 cases), 47.14% (2,502 cases), respectively.

Table 1 showed the descriptive statistics for the baseline variables in terms of SDNN quartiles. The group with a lower SDNN value tended to be older, with an increased proportion of women, faster heart rate, and higher systolic blood pressure at first admission. In the medical history, the prevalence of diabetes mellitus increased significantly in the group with a low SDNN value, and there was a significant difference between groups (*p* < 0.0001).

**Table 1:** Baseline characteristics for study sample by SDNN quartile (*n* = 5,308).

**Characteristics**	**SDNN Quartile**				
**Category**	**1**	**2**	**3**	**4**	***P*-value**
*N*	1,364	1,291	1,346	1,307	
Range, ms	<88	88–105.5	105.5–26	>126	
Age (years)	63.18 ± 10.86	61.58 ± 10.5	60.07 ± 10.36	59.62 ± 11.15	<0.0001
Male, *n* (%)	838 (61.44)	870 (67.39)	956 (71.03)	1,005 (76.83)	<0.0001
**Cigarette smoking**, ***n*** **(%)**					
Never	775 (56.82)	667 (51.67)	639 (47.47)	576 (44.07)	<0.0001
Occasionally	57 (4.18)	48 (3.72)	55 (4.09)	64 (4.90)	
Current	360 (26.39)	417 (32.30)	507 (37.67)	494 (37.80)	
Former	172 (12.61)	159 (12.32)	145 (10.77)	173 (13.24)	
**Alcohol consumption**, ***n*** **(%)**					
Never	822 (60.26)	680 (52.67)	676 (50.22)	641 (49.04)	<0.0001
Occasionally	266 (19.50)	303 (23.47)	343 (25.48)	313 (23.95)	
Current	180 (13.20)	220 (17.04)	239 (17.76)	259 (19.82)	
Former	96 (7.04)	88 (6.82)	88 (6.54)	94 (7.19)	
BMI, kg/m^2^, mean (SD)	24.71 ± 3.33	24.88 ± 3.37	25.09 ± 3.15	24.8 ± 3.11	0.0100
Waistline	85 (79–95)	87 (79–95)	87 (80–96)	87 (79–95)	0.3528
Heart rate on admission, bpm, mean (SD)	76 (70–82)	76 (70–80)	75 (68–80)	72 (65–79)	<0.0001
**Blood pressure on admission (mmHg)**					
Right systolic BP, mmHg, mean (SD)	150 (138–166)	150 (134–165)	150 (135–165)	148 (133–163)	0.0049
Right diastolic BP, mmHg, mean (SD)	90 (80–97)	87 (80–99)	89 (80–98)	88 (80–97)	0.3011
Left systolic BP, mmHg, mean (SD)	150 (136–162.5)	149 (135–162)	149 (135–162)	145 (132–160)	0.0033
Left diastolic BP, mmHg, mean (SD)	86 (80–95)	86 (80–96)	86 (80–96)	85 (80–95)	0.3953
NIHSS score	2 (1–4)	2 (1–4)	2 (1–4)	2 (1–3)	<0.0001
**Medical history**, ***n*** **(%)**					
Previous Stroke/TIA	352 (25.81)	280 (21.69)	279 (20.73)	304 (23.26)	0.0102
Previous heart disease	170 (12.46)	140 (10.84)	133 (9.88)	125 (9.56)	0.0657
Hypertension	872 (63.93)	799 (61.89)	836 (62.11)	789 (60.29)	0.2862
Diabetes mellitus	398 (29.18)	289 (22.39)	284 (21.10)	246 (18.82)	<0.0001
Dyslipidemia	99 (7.26)	111 (8.60)	110 (8.17)	113 (8.65)	0.5273
mRS score before onset					0.9001
0	1,031 (75.59)	999 (77.38)	1,042 (77.41)	995 (76.13)	
1	241 (17.67)	208 (16.11)	222 (16.49)	232 (17.75)	
2	54 (3.96)	55 (4.26)	53 (3.94)	53 (4.06)	
3	24 (1.76)	15 (1.16)	19 (1.41)	17 (1.30)	
4	13 (0.95)	14 (1.08)	9 (0.67)	8 (0.61)	
5	1 (0.07)	0 (0.00)	1 (0.07)	2 (0.15)	
**Medication history**					
Antiplatelet agents	218 (15.98)	195 (15.10)	190 (14.12)	200 (15.30)	0.5975
Anticoagulant agents	1 (0.07)	3 (0.23)	2 (0.15)	3 (0.23)	0.7149
Stains	133 (9.75)	119 (9.22)	105 (7.80)	136 (10.41)	0.1205
Hypoglycemic drugs	323 (23.68)	222 (17.20)	228 (16.94)	186 (14.23)	<0.0001
Antioxidant against lipid peroxidation	5 (0.37)	5 (0.39)	2 (0.15)	3 (0.23)	0.6080
Antihypertensive drugs	633 (46.41)	551 (42.68)	579 (43.02)	546 (41.78)	0.0801
β-blockers	41 (3.01)	30 (2.32)	21 (1.56)	25 (1.91)	0.0641
**Inpatient therapy**					
Dual antiplatelet therapy	186 (13.64)	198 (15.34)	196 (14.56)	165 (12.62)	0.2160
Anti-platelet therapy	1,332 (97.65)	1,267 (98.14)	1,319 (97.99)	1,283 (98.16)	0.7704
Anticoagulation treatment	85 (6.23)	84 (6.51)	72 (5.35)	55 (4.21)	0.0457
Stains	1,307 (95.82)	1,240 (96.05)	1,280 (95.10)	1,250 (95.64)	0.6635
Antioxidant treatment	226 (16.57)	198 (15.34)	211 (15.68)	203 (15.53)	0.8253
Hypoglycemic treatment	436 (31.96)	327 (25.33)	302 (22.44)	256 (19.59)	<0.0001
Antihypertensive treatment	690 (50.59)	603 (46.71)	611 (45.39)	598 (45.75)	0.0261
CCB	522 (38.27)	473 (36.64)	475 (35.29)	446 (34.12)	0.1362
ACEI or ARB	251 (18.40)	225 (17.43)	222 (16.49)	236 (18.06)	0.5816
Diuretic	29 (2.13)	28 (2.17)	28 (2.08)	41 (3.14)	0.2232
β-blockers	62 (4.55)	39 (3.02)	34 (2.53)	32 (2.45)	0.0055
α-blockers	2 (0.15)	3 (0.23)	1 (0.07)	1 (0.08)	0.6468
TOAST					0.0205
Large artery atherosclerosis;	379 (27.79)	311 (24.09)	307 (22.81)	315 (24.10)	
Cardioembolism	21 (1.54)	19 (1.47)	16 (1.19)	22 (1.68)	
Small vascular occlusion	307 (22.51)	329 (25.48)	346 (25.71)	309 (23.64)	
Other determination	27 (1.98)	18 (1.39)	15 (1.11)	9 (0.69)	
Undetermined	630 (46.19)	614 (47.56)	662 (49.18)	652 (49.89)	

*SDNN, the standard deviation of all N-N intervals; BMI, body mass index; SD, standard deviation; BP, blood pressure; NIHSS, National Institute of Health stroke scale; TIA, transient ischemic attack; mRS, modified Rankin scale; CCB, calcium channel blockers; ACEI, angiotensin converting enzyme inhibitors; ARB, angiotensin receptor blockers; TOAST, Trial of Org 10172 in Acute Stroke Treatment*.

With a gradual increase of SDNN, the rate of neurological disability in patients with TIA or minor stroke decreased significantly 90 days after stroke. Compared with the lowest SDNN quartile, findings for the other three groups were as follows, [OR in group Q2 after correction, 0.659; 95% confidence interval (CI), 0.482–0.900; *p* = 0.0088; OR of group Q3, 0.662; 95% CI, 0.478–0.916; *p* = 0.0127; OR of group Q4, 0.441; 95% CI, 0.305–0.639; *p* < 0.0001]. With an increasing SDNN, stroke recurrence showed a decreasing trend. The recurrence rate of patients of the two higher quartiles of SDNN was significantly reduced at 90 days (HR of Q3 group after correction, 0.732; 95% CI, 0.539–0.995; *p* = 0.0461; HR of Q4 group after correction, 0.528; 95% CI, 0.374–0.745; *p* = 0.0003). No clear association was found between SDNN and cardiovascular death (Table 2). In addition, no association was found between RMSSD and 90-day neurological disability, recurrent stroke, or cardiovascular death (Table 3).

**Table 2:** Correlation of SDNN with the 90-day prognosis of ischemic stroke.

**Outcomes**	**SDNN**	**Events *n* (%)**	**Model 1 unadjusted**		**Model 2 adjusted**	
			**OR/HR (95% CI)**	***P-*value**	**OR/HR (95% CI)**	***P-*value**
Disability (mRS, 3–5)	Q1	123 (9.16)	Reference		Reference	
	Q2	73 (5.69)	0.599 (0.444–0.809)	0.0008	0.659 (0.482–0.900)	0.0088
	Q3	65 (4.88)	0.509 (0.373–0.694)	<0.0001	0.662 (0.478–0.916)	0.0127
	Q4	43 (3.32)	0.341 (0.239–0.487)	<0.0001	0.441 (0.305–0.639)	<0.0001
Recurrent stroke	Q1	104 (7.62)	Reference		Reference	
	Q2	84 (6.51)	0.846 (0.635–1.128)	0.2555	0.878 (0.657–1.173)	0.3779
	Q3	71 (5.27)	0.683 (0.505–0.924)	0.0133	0.732 (0.539–0.995)	0.0461
	Q4	49 (3.75)	0.481 (0.342–0.676)	<0.0001	0.528 (0.374–0.745)	0.0003
Cardiovascular death	Q1	5 (0.37)	Reference		Reference	
	Q2	3 (0.23)	0.631 (0.151–2.638)	0.5278	0.654 (0.154–2.785)	0.5658
	Q3	3 (0.22)	0.606 (0.145–2.536)	0.4930	0.668 (0.156–2.852)	0.5858
	Q4	2 (0.15)	0.417 (0.081–2.148)	0.2955	0.514 (0.097–2.730)	0.4349

*Model 1: unadjusted. Model 2: adjusted for age, sex, smoking, alcohol consumption, body mass index, heart rate on admission, systolic and diastolic blood pressures on admission, NIHSS (National Institute of Health stroke scale) score, medical history, mRS score before onset, dual antiplatelet therapy, and stroke etiology (TOAST). HR can be interpreted as the comparison between quartiles 2, 3, and 4 with quartile 1 (reference). OR/HR can be interpreted as the comparison between quartiles 2, 3, and 4 with quartile 1 (reference)*.

*SDNN, the standard deviation of all N-N intervals; OR, odds ratio; HR, hazard ratio; CI, confidence interval; mRS, modified Rankin scale*.

*SDNN: Q1 < 88 ms; Q2: 88–105.5 ms; Q3: 105.5–126 ms; Q4 >126 ms*.

**Table 3:** Correlation of RMSSD with the 90-day prognosis of ischemic stroke.

**Outcomes**	**RMSSD**	**Events *n* (%)**	**Model 1 unadjusted**		**Model 2 adjusted**	
			**OR/HR (95% CI)**	***P-*value**	**OR/HR (95% CI)**	***P-*value**
Disability (mRS, 3–5)	Q1	88 (6.31)	Reference		Reference	
	Q2	68 (5.33)	0.836 (0.604–1.158)	0.2818	0.886 (0.632–1.243)	0.4841
	Q3	65 (4.93)	0.770 (0.554–1.071)	0.1202	0.838 (0.595–1.180)	0.3113
	Q4	83 (6.57)	1.044 (0.766–1.423)	0.7858	1.059 (0.765–1.465)	0.7298
Recurrent stroke	Q1	96 (6.79)	Reference		Reference	
	Q2	65 (5.06)	0.737 (0.538–1.009)	0.0572	0.745 (0.543–1.022)	0.0683
	Q3	74 (5.56)	0.814 (0.601–1.102)	0.1833	0.830 (0.612–1.126)	0.2317
	Q4	73 (5.72)	0.837 (0.617–1.135)	0.2524	0.855 (0.629–1.161)	0.3146
Cardiovascular death	Q1	7 (0.50)	Reference		Reference	
	Q2	0 (0.00)	-	-	-	-
	Q3	1 (0.08)	0.151 (0.019–1.231)	0.0775	0.142 (0.017–1.165)	0.0692
	Q4	5 (0.39)	0.791 (0.251–2.491)	0.6882	0.633 (0.196–2.042)	0.4442

*Model 1: unadjusted. Model 2: adjusted for age, sex, smoking, alcohol consumption, body mass index, heart rate on admission, systolic and diastolic blood pressures on admission, NIHSS (National Institute of Health stroke scale) score, medical history, mRS score before onset, dual antiplatelet therapy, and stroke etiology (TOAST). OR/HR can be interpreted as the comparison between quartiles 2, 3, and 4 with quartile 1 (reference)*.

*RMSSD, the square root of the mean squared differences of successive N-N intervals; OR, odds ratio; HR, hazard ratio; CI, confidence interval; mRS, modified Rankin scale*.

*RMSSD: Q1 < 20 ms; Q2: 20–27 ms; Q3: 27–38 ms; Q4 >38 ms*.

## Discussion

In the time domain measurement of HRV, SDNN reflects the overall condition of the autonomic nerve system, and a decrease of the SDNN usually indicates a relative superiority of the sympathetic nerve in the autonomic nervous system (24). Aging (25), female sex (25, 26), increased blood pressure, and diabetes mellitus (26, 27) all showed the characteristics of relative sympathetic nerve superiority in the autonomic nervous system, and thus with a decreased SDNN. This is consistent with our baseline data analysis results (Table 1). In this study, we found that with an increasing SDNN, the rate of neurological dysfunction in patients at 90 days after ischemic stroke decreased significantly. The recurrence rate of 90-day stroke was significantly reduced for participants in the two higher quartiles of SDNN.

Previous studies have shown that age, diabetes mellitus, and NIHSS scores are predictors of 90-day neurological disability (28, 29). But in this study, after correcting for confounding factors, we found that with an increasing SDNN, the rate of neurological dysfunction in patients 90 days after ischemic stroke decreased significantly. After the acute phase, patients with autonomic nerve system dysfunction need more help in their daily rehabilitation tasks (30). Poor adaptability of the cardiac autonomic nerve system in different rehabilitation training activities and poor rehabilitation dependence impact the rehabilitation effects (30, 31). At the same time, autonomic nerve system dysfunction is not only associated with overall cognitive function, processing speed, executive function, and poor retrospective memory performance in patients (32, 33) but also associated with post-stroke depression (34). All of these might have a negative impact on the patients' positive initiative in rehabilitation training and their ability to follow the rehabilitation regimens, resulting in unsatisfactory rehabilitation results.

According to previous studies, age, blood pressure and diabetes mellitus were all risk factors for recurrence of TIA and minor stroke (35, 36). However, after adjusting for risk factors such as age, blood pressure, and diabetes mellitus, SDNN was still significantly correlated with stroke recurrence, suggesting that autonomic dysregulation was associated with stroke recurrence. There is a balance between the sympathetic and parasympathetic nervous systems, which is important for regulating cerebral blood flow. Dysfunction of the autonomic nervous system after stroke aggravated secondary brain injury through changes in hemodynamics (37) and non-hemodynamic factors. Changes in hemodynamics, such as increased blood pressure variability, impaired brain autoregulation, and cardiovascular complications, lead to secondary brain injury. Non-hemodynamic factors such as the production of inflammatory factors (38), hyperglycemia, and increased blood-brain barrier permeability (39), coagulation factor activation, and platelet activation (40, 41) also cause secondary brain damage. These all increase the risk of further vascular events, such as myocardial infarction, recurrent stroke, and deep vein thrombosis (40). In animal experiments, chronic stress increased sympathetic nerve activity to increase the heart rate of mice. It was found that vascular endothelial function was damaged and oxidative stress in the blood vessels and brain as well as the susceptibility to cerebral ischemia were increased (42), consequently increasing the area of brain injury. Lowering the heart rate can restore vascular endothelial function, reduce oxidative stress, increase capillary density and collateral circulation (43), protect ischemic brain injury (43), and reduce stroke volume (44). The above mechanisms may explain our findings, that is, sympathetic hyperexcitation leads to poor neurological outcomes and stroke recurrence, while sympathetic suppression leads to favorable neurological outcomes and a reduction in stroke recurrence.

Previous evidence had shown that 24-h SDNN was strongly associated with all-cause mortality (45). Low HRV predicted increased mortality, and the association could not be attributed to cardiovascular risk factors or underlying disease (23). The cardiac complications resulting from autonomic dysfunction in stroke patients were 2–6% of the total mortality rate 90 days after acute ischemic stroke (46). In our study, both SDNN and RMSSD were not associated with 90-day vascular death in patients. This is different from previous studies, which may be due to the study population difference. The study population we selected were TIA and minor stroke patients with an NIHSS score ≤5, with mild clinical symptoms, and a total mortality rate of 0.24%.

SDNN reflects the overall autonomic function, including sympathetic and parasympathetic activity, while RMSSD only reflects parasympathetic activity (13). The parasympathetic effect is transient, while there is a long period of sympathetic excitation after ischemic stroke (47). Therefore, considering that 90-day neurological dysfunction and stroke recurrence are both associated with sympathetic hyperexcitability after ischemic stroke, reducing sympathetic activity may improve the prognosis of ischemic stroke after 90 days. Vagal nerve stimulation (VNS) was also shown to reduce infarct volume and improve neurological outcome at 1 day after acute ischemic stroke in middle cerebral artery occlusion rats (48). The mechanism of protection with VNS may involve a reduction in extracellular glutamate and reduced excitotoxicity during cerebral ischemia, and/or a reduction in inflammation and release of norepinephrine. Parasympathetic activation also increases cerebral blood flow and enhance neurogenesis. However, parasympathetic activation is an invasive technique, which limits its use in acute stroke treatment (49). Beta blockade (50), statin (51), external counter pulsation (52), tele-acupuncture (53) have all been reported to modulate autonomic nervous dysfunction although more research is needed to confirm these findings.

## Limitations

Our research has some limitations. Firstly, CNSR-III is a prospective clinical registry study of ischemic stroke or TIA nationwide based on etiological classification, imaging, and biological markers. It is not a specific study on the correlation between HRV and stroke prognosis. Second, patients received 24-h Holter during the acute period of hospitalization for stroke, and we did not conduct statistics on the interval between the examination and stroke occurrence. Third, in this study, we mainly analyzed the correlation between autonomic nerve system function and short-term prognosis of patients with TIA and minor ischemic stroke. Considering that TIA has no lesions and the lesions of minor stroke are relatively small, the localization of the lesions has not been evaluated yet. The relationship between stroke location and HRV need be investigated.

## Conclusion

This study shows that autonomic nerve system dysfunction (sympathetic hyperexcitability and/or decreased parasympathetic activity) is an adverse factor for 90-day neurological prognosis and stroke recurrence after TIA and minor stroke. Regulating autonomic nerve system function may be a potential new target for improving the 90-day prognosis in these patients and is worthy to be further investigated.

## Data Availability Statement

The original contributions presented in the study are included in the article/supplementary material, further inquiries can be directed to the corresponding author.

## Ethics Statement

The studies involving human participants were reviewed and approved by Beijing Tiantan Hospital affiliated to Capital Medical University (IRB Approval Number: KY2015-001-01) and all participating centers. The patients/participants provided their written informed consent to participate in this study. Written informed consent was obtained from the individual(s) for the publication of any potentially identifiable images or data included in this article.

## Author Contributions

CL conceived the study and wrote the first draft of the paper. YP and MW analyzed the data. XM and ZL critically edited the manuscript. YW supervised the study. All authors contributed to the article and approved the submitted version.

## Conflict of Interest

The authors declare that the research was conducted in the absence of any commercial or financial relationships that could be construed as a potential conflict of interest.
